# Rotating-Coil Measurement System for Small-Bore-Diameter Magnet Characterization

**DOI:** 10.3390/s22218359

**Published:** 2022-10-31

**Authors:** Anna Lauria, Pasquale Arpaia, Marco Buzio, Antonio Gilardi, Marco Parvis, Mariano Pentella, Lucia Sabbatini, Enzo Simoni, Alessandro Vannozzi

**Affiliations:** 1 Department of Electronics and Telecommunications, Polytechnic of Turin, 10129 Turin, Italy; 2 Technology Department, European Organization for Nuclear Research (CERN), 1211 Geneva, Switzerland; 3 Department of Electrical Engineering and Information Technology, University of Naples Federico II, 80131 Naples, Italy; 4 SLAC National Accelerator Laboratory, Menlo Park, CA 94025, USA; 5 Frascati National Laboratory, National Institute for Nuclear Physics (INFN), 00044 Frascati, Italy; 6 Faculté de Mesures Physiques, Institut Universitaire de Technologie, Université Savoie Mont-Blanc, 74000 Annecy, France

**Keywords:** rotating coil, quadrupole magnet, PCB coil, magnetic measurement

## Abstract

Rotating-coil measurement systems are widely used to measure the multipolar fields of particle accelerator magnets. This paper presents a rotating-coil measurement system that aims at providing a complete data set for the characterization of quadrupole magnets with small bore diameters (26 mm). The PCB magnetometer design represents a challenging goal for this type of transducer. It is characterized by an aspect ratio 30% higher than the state of the art, imposed by the reduced dimension of the external radius of the rotating shaft and the necessity of covering the entire magnet effective length (500 mm or higher). The system design required a novel design for the mechanical asset, also considering the innovation represented by the commercial carbon fiber tube, housing the PCB magnetometer. Moreover, the measurement system is based primarily on standard and commercially available components, with simplified control and post-processing software applications. The system and its components are cross-calibrated using a stretched-wire system and another rotating-coil system. The measurement precision is established in a measurement campaign performed on a quadrupole magnet characterized by an inner bore diameter of 45 mm.

## 1. Introduction

Magnets are one of the main components in particle accelerators, where they are used to steer and focus the particle beam along the nominal trajectory. Dipole magnets are used for steering/bending the particle beam, whereas quadrupole magnets focus and squeeze the beam. In this context, field quality plays an essential role [1]. Field errors determine beam perturbations, resulting in emittance growth and in a beam unsuitable for the experiments [2,3].

The field quality, expressed in terms of the strength and phase of the main field component, plus the unavoidable higher error harmonics (see Section 2.1), is a requirement for magnet designers. Assessment of the field quality is performed through accurate field measurements, necessary to determine the presence of construction errors, coil deformations due to electromagnetic forces, dynamical effects such as eddy currents in the bulk of the yoke, ferromagnetic hysteresis, and iron saturation. Complementary methods exist to perform magnetic measurements [4], each one optimized for a determined quantity of interest, such as integrated field, field harmonics, or time evolution of the field in the magnet gap.

One of the most relevant techniques to test accelerator magnets is the *rotating-coil* method [5,6,7,8,9], also called the harmonic coil. The rotating coil consists of a cylindrical shaft containing a certain number of induction coils, i.e., field-sensing transducers based on Faraday’s law of induction, which are rotated around the longitudinal axis of the magnet. This method generally provides field error measurements with repeatability up to 1 ppm [10,11,12].

In the context of an ongoing collaboration [13] between the European Organization for Nuclear Research (CERN) and the Istituto Nazionale di Fisica Nucleare (INFN) of Frascati (Italy), our goal is the development of a new small-radius rotating-coil measurement system to test the field quality of quadrupoles with small-diameter bores for the European project EuPRAXIA (European Plasma Research Accelerator with eXcellence In Applications) [14]. The EuPRAXIA project aims at designing an accelerator based on the plasma wakefield technique, which can deliver a 5 GeV electron beam with simultaneously high charge, low emittance, and low energy spread to users. Its foreseen electron energy range and performance goals will enable versatile applications in various domains, e.g., compact free-electron lasers (FELs) or sources for medical imaging and positron generation, table-top test beams for particle detectors, as well as deeply penetrating X-ray and gamma-ray sources for material testing. This new class of plasma-based accelerators is designed to be the stepping stone to possible future plasma-based facilities, such as linear colliders at the high-energy physics (HEP) energy frontier.

Driving plasma accelerator beams requires a particular beam manipulation and associated magnetic elements. The most critical components of this machine are the quadrupole magnets. The features of the quadrupole magnet prototype BTF7, which has been tested at CERN with the prototype rotating-coil system, are summarized in Table 1.

The characterization of small-bore magnets is challenging. The sag of a cylindrical tube such as the measurement coil shaft, having diameter *D* and length *L*, simply supported at both ends, is given in the first approximation by the following expression [15]:

(1) $$d=\frac{5}{\alpha }\rho g\frac{L^{4}}{ED^{2}}$$

where ρ is its average volume mass density, *E* its effective Young’s modulus, and the numerical coefficient α is equal to 24 for a round bar and 48 for a thin cylindrical tube, irrespective of its thickness, within an error less than 10% when the ratio of thickness to diameter is less than 5%. Increasing the aspect ratio $\lambda =\frac{L^{4}}{D^{2}}$ leads very rapidly to a high sag and undesired flexural oscillations during the rotation, which affect the accuracy of the measured harmonics [16]. The impact of such mechanical imperfections can be mitigated by the coil bucking technique [4], which, however, requires very tight tolerances on the geometry of the coils. In addition, the small size of the rotating shaft and the constraints on the density of coil turns, which can vary typically between 100 (for multi-layer PCBs) and 400 turns/mm${}^{2}$ (for multi-wire flat cables), reduce the magnetometer coil sensitivity, especially on the higher-order harmonics.

An example of a small rotating-coil magnetometer is presented in [17], which describes a ø19 mm and 500 mm long rotating-coil probe ($\lambda =173\;m^{2}$) built to test permanent magnet quadrupoles for CERN’s Linac4. The proposed design, based on a machined stack of 80 PCB layers, proved ineffective in rejecting spurious harmonics due to mechanical imperfections. In [18], a ø8 mm rotating-coil system to test CLIC protoytpe magnets with an aperture of less than ø10 mm, necessary to obtain the very high gradient of 200 T/m, is discussed. This extremely small size was achieved thanks to the choice of synthetic sapphire as the support material, and the short required length of only 150 mm ($\lambda =8\;m^{2}$). Synthetic sapphire (Al_2_O_3_) has an exceptionally high $\frac{E}{\rho }$ ratio of around $110\times 10^{12}\;m^{2}s^{-2}$, i.e., almost ten times as high as fiberglass. This material can be sintered and polished within tolerances of a few μm, which makes it an ideal candidate for high-precision coil supports. Unfortunately, it has a high cost and it cannot be produced in large sizes.

Since the sag of a simply supported shaft grows with the fourth power of its length, coils significantly shorter than the magnet are commonly used to characterize magnets whose length exceeds one or two meters. For example, a rotating-coil system was developed at CERN for measuring the local magnetic flux density of quadrupoles for the HL-LHC Insertion Region (IR) [19], characterized by a large bore diameter (around 90 mm). Short coils were also designed to reject pseudo-multipole components in short magnets dominated by the fringe field [20].

The geometric requirements imposed by the EuPRAXIA quadrupole magnets exceed the performance of all these systems. This paper aims to present the design, calibration, and validation of a prototype ø26 mm diameter rotating-coil system to be used for the measurement of the EuPRAXIA quadrupole magnets. The smallest foreseen bore diameter is 30 mm, which applies to the final focus quadrupoles and drives the requirement for the rotating-coil shaft diameter. The corresponding shaft aspect ratio is $\lambda =44\;m^{2}$, which is challenging and requires a substantial improvement over our previous ø19 mm design. Another important goal is to simplify the design as much as possible and use low-cost, off-the-shelf components and open-source software packages, so as to widen the accessibility of this technology. The requirements for the proposed rotating-coil system, which will be followed by a more complete final system at a later stage, are summarized in Table 2.

## 2. Background Theory

### 2.1. Field Harmonics Theory Fundamentals

The air gap of a magnet can be modeled as a region free of current sources. Considering a cylindrical volume, coaxial with the gap, sufficiently extended so that the field at both ends can be considered to vanish, the integral magnetic field density satisfies the 2D Laplace equation and can be described by a complex-valued harmonic field expansion in a circular domain of radius $r_{0}$, also called the reference radius [4]. For $\underline{z}=x+iy$,

(2) $$\underline{B}\left(\underline{z}\right)=B_{y}\left(x,y\right)+iB_{x}\left(x,y\right)=\sum_{n=1}^{\infty }{\underline{C}}_{n}\left(r_{0}\right){\left(\frac{\underline{z}}{r_{0}}\right)}^{n-1}$$

where $B_{y}$ and $B_{x}$ represent the vertical and horizontal field components as a function of the position, ${\underline{C}}_{n}=B_{n}+iA_{n}$ is the *n*-th complex harmonic coefficient, and $B_{n}$ and $A_{n}$ are the normal and skew harmonic coefficients, respectively, expressed in tesla at the reference radius $r_{0}$. The reference radius defines the limit of the desired good field region, which is that occupied by the particle beam, and is usually set at 2/3 of the magnet aperture radius, where the field errors remain within the tolerance values. Each value of the integer *n* corresponds to a specific flux distribution generated by an ideal 2n-pole magnet. In most practical cases, magnets are designed to provide only one main harmonic component *m*. Higher-order harmonics, arising from imperfections and manufacturing tolerances, are used as indications of the field quality. For an ideal quadrupole magnet (*n* = 2), the main field component is given by

(3) $$\begin{array}{cc} B_{x}\left(x,y\right) & =\frac{1}{r_{0}}\left(B_{2}\left(r_{0}\right)y+A_{2}\left(r_{0}\right)x\right)\\ B_{y}\left(x,y\right) & =\frac{1}{r_{0}}\left(B_{2}\left(r_{0}\right)x-A_{2}\left(r_{0}\right)y\right). \end{array}$$

The amplitude of the two components varies linearly with the distance from the origin. The coefficients $B_{n}$ and $A_{n}$ of the series expansion determine the shape of the magnetic field lines. A quadrupole magnet in which only the coefficient $B_{2}$ is non-zero and the skew coefficient is $A_{2}=0$ is called a normal magnet and the field components can be expressed as

(4) $$\begin{array}{cc} B_{x}\left(x,y\right) & =Gy\\ B_{y}\left(x,y\right) & =Gx \end{array}$$

where $G=B_{2}/r_{0}$ is the field gradient, expressed in Tm${}^{-1}$.

The origin of the reference system is referred to as the magnetic center, and it represents where all multipolar field components of order n≤2 are above zero. The expansion coefficients $B_{n}$ and $A_{n}$ are a function of the excitation current level. In most practical cases, being interested in the shape of the field rather than its absolute magnitude, the harmonic coefficients are normalized with respect to the main field component $B_{M}$ ($B_{2}$ for a quadrupole magnet), and expressed in units of $10^{-4}$,

(5) $$c_{n}\left(r_{0}\right)=10^{4}\frac{C_{n}\left(r_{0}\right)}{B_{M}\left(r_{0}\right)}=10^{4}\left(\frac{B_{n}\left(r_{0}\right)}{B_{M}\left(r_{0}\right)}+i\frac{A_{n}\left(r_{0}\right)}{B_{M}\left(r_{0}\right)}\right)=b_{n}+ia_{n}.$$

This choice arises from the typical tolerance values of accelerator magnets, where the harmonics are in the order of 0.01% of the main field component. There are different applications and experiments for which having a precise magnetic characterization is crucial [21,22,23,24].

### 2.2. Rotating-Coil Magnetometers

Rotating-coil magnetometers belong to the family of induction coil sensors, where the magnetic field is measured through the flux variation induced in a sensing coil (also called a pick-up coil). According to Faraday’s law of induction, the voltage at the terminal of a sensing coil is proportional to the rate of change of the magnetic flux:

(6) $$U\left(t\right)=-\frac{d\Phi (t)}{dt},$$

where Φ is the total magnetic flux linked with the coil. The measurement is generally performed by rotating the shaft in a static field.

**Figure 1:**
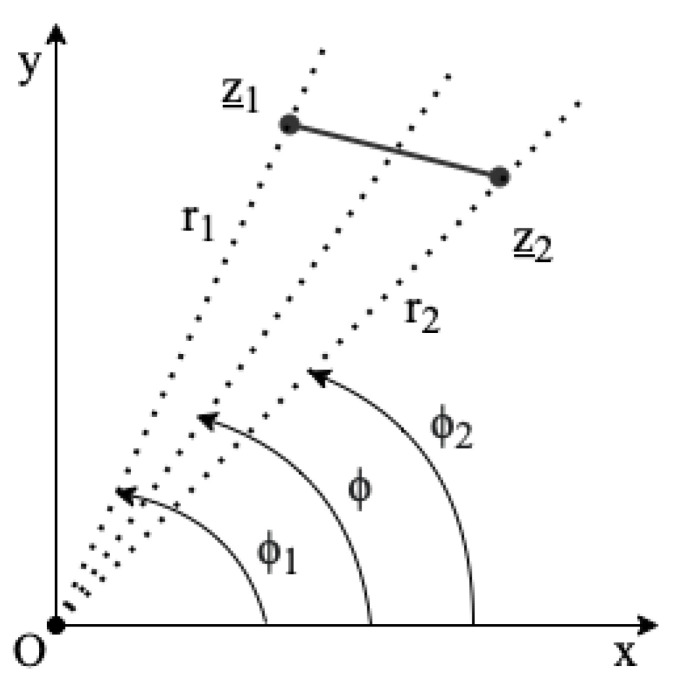
Naming convention for angles and radii in the ideal case of a point-link coil winding pack.

Rotating-coil magnetometers are composed of a certain number of induction coils, generally long rectangular loops of wire, constrained in width by the magnet aperture diameter. Considering a point-like winding case, the geometry of an ideal induction coil can be described by the complex points ${\underline{z}}_{1}$ and ${\underline{z}}_{2}$ shown in Figure 1, representing the location of the conductor turns in the magnet transverse plane. The origin of the reference system does not need to be necessarily coincident with the magnetic axis of the magnet under test. Considering the cross-section of a multi-turn and multi-layer coil, all the winding turns can be assumed as perfectly packed together on the central positions ${\underline{z}}_{1}$ and ${\underline{z}}_{2}$ in the analysis, with negligible uncertainty [25].

Given an induction coil with *N* turns, a total area $A_{c}$, and length *L*, the linked magnetic flux can be calculated as

(7) $$\begin{array}{cc} \Phi & =N\int_{A_{c}}B\;da=N\int_{0}^{L}\int_{{\underline{z}}_{1}}^{{\underline{z}}_{2}}\left(B_{y}\left(x,y\right)dx-B_{x}\left(x,y\right)dy\right)\;d\underline{z}\\ \; & =NL\int_{{\underline{z}}_{1}}^{{\underline{z}}_{2}}\left(B_{y}\left(x,y\right)dx-B_{x}\left(x,y\right)dy\right) \end{array}$$

where ${\underline{z}}_{1}=r_{1}e^{i\phi_{1}}$ and ${\underline{z}}_{2}=r_{2}e^{i\phi_{2}}$. From (2) and considering

(8) $$\int_{{\underline{z}}_{1}}^{{\underline{z}}_{2}}\underline{B}\left(\underline{z}\right)d\underline{z}=\int_{{\underline{z}}_{1}}^{{\underline{z}}_{2}}\left(B_{y}\left(x,y\right)dx-B_{x}\left(x,y\right)dy\right)+i\int_{{\underline{z}}_{1}}^{{\underline{z}}_{2}}\left(B_{y}\left(x,y\right)dy+B_{x}\left(x,y\right)dx\right)$$

Equation (7) becomes

(9) Φ(ϕ)=NLRe∫z_1z_2B_(z_)dz_=NLRe∫z_1z_2∑n=1∞Cn(r0)(z_r0)n−1dz_=Re∑n=1∞NLnr0(n−1)C_n(r0)(z_2n−z_1n)=Re∑n=1∞C_nS_neinϕ

where ${\underline{S}}_{n}$ are complex sensitivity coefficients, representing geometric factors and related to the position of the turns in the reference frame. The origin of the reference frame is set as coincident with the shaft rotation axis. The sensitivity coefficients have the physical units of m${}^{\left(n+1\right)}$, and a magnitude scaling exponentially with *n*. These coefficients are usually normalized to $r_{0}^{\left(n-1\right)}$ to express them in m${}^{2}$ and have numbers numerically comparable:

(10) $${\underline{S}}_{n}\left(r_{0}\right)=\frac{NL}{nr_{0}^{\left(n-1\right)}}\left({\underline{z}}_{2}^{n}-{\underline{z}}_{1}^{n}\right)=\frac{NL}{nr_{0}^{\left(n-1\right)}}\left(r_{2}^{n}e^{in(\phi_{2}-\phi )}-r_{1}^{n}e^{in(\phi_{1}-\phi )}\right).$$

The complex field harmonic coefficients $C_{n}\left(r_{0}\right)$ are calculated from the measured flux as

(11) $${\underline{C}}_{n}\left(r_{0}\right)=B_{n}\left(r_{0}\right)+iA_{n}\left(r_{0}\right)=\frac{{\underline{\Psi }}_{n}}{{\underline{S}}_{n}}r_{0}^{n-1}$$

where ${\underline{\Psi }}_{n}$ are the complex coefficients of the Discrete Fourier Transform (DFT) of the measured flux,

(12) $${\underline{\Psi }}_{n}=\frac{2}{N}\sum_{k=1}^{N}\Phi_{k}e^{-2\pi i\frac{k}{N}}.$$

## 3. Proposal

### 3.1. PCB-Based Induction Coil Magnetometer

The EuPRAXIA project application represents a quite extreme case for the proposed typology of transducers, planned to be installed in a rotating shaft with an inner diameter of 26 mm and an overall length of 640 mm. The shaft dimensions allow coverage of the entire magnet length of 440 mm and its fringe field region.

The aspect ratio of the proposed PCB coil shaft, defined as $\frac{L^{4}}{D^{2}}$, is around 250, while being around 170 for the ø19 mm probe and around 10 for the ø8 mm probe, introduced in Section 1. Since mechanical imperfections exist even for smaller aspect ratios, and given the intention of avoiding expensive materials (such as synthetic sapphire), the much higher aspect ratio for the proposed ø26 mm rotating-coil system required a novel design for the mechanical asset. Moreover, also the innovation represented by the commercial carbon fiber tube housing the PCB magnetometer had an influence in the support structure design.

The realization of such a small-radius rotating-coil system significantly impacts the size of the coil array and, in particular, the geometric width of each coil and their area. A reduced area means reduced sensitivity to the field harmonic components. The proposed coil array design aims at counterbalancing this effect.

The adopted technology for the sensing coils is based on the Printed Circuit Board (PCB) technology, a well-known alternative to the traditional wound coils [17,18,19]. The PCB technology is characterized by the high precision of copper track positioning (± 2 μm). The possibility to achieve higher control over the coil sensitivity and the careful calibration process allow gradient measurement with accuracy up to the 0.1% level [12].

**Figure 2:**
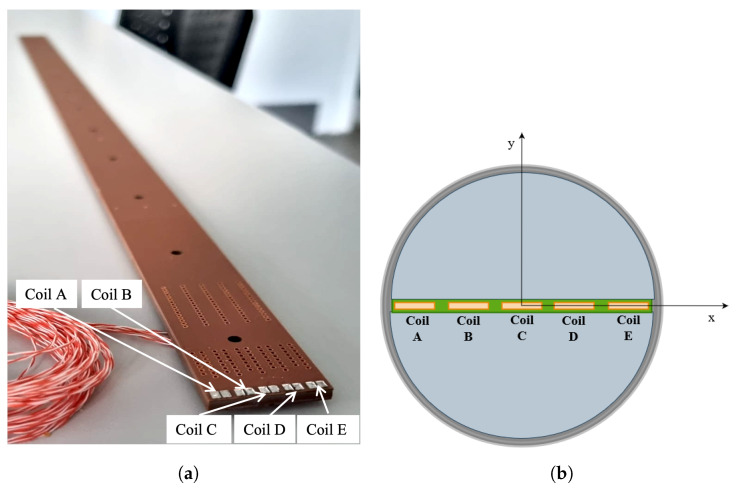
(**a**) The PCB array magnetometer with five identical coils in radial configuration; each layer has eight turns. The drawing is not to scale. (**b**) Schematic representation of the rotating PCB with 5 radial coils.

The proposed PCB coil array was produced at CERN. A PCB array of 5 identical coils, each in the shape of a rectangular and planar spiral, positioned in a radial configuration, was designed. The problem of the geometric constraints was addressed by designing the PCB array with the highest possible number of PCB layers in the available space to increase the turn density and the coil sensitivity. In the adopted design, the PCB layers are connected in series with metallic holes drilled in the compressed stack of layers. The PCB layers have the same nominal surface, and the connections of the pads are placed close together to minimize the spurious surface of wires between them. A picture of the produced PCB coil array and a schematic cross-section representation of the PCB array inside the rotating shaft are shown in Figure 2.

The design parameters of the PCB array are reported in Table 3. The main manufacturing parameters of the PCB are reported in Table 4. These parameters are unusual [26] and could be achieved only by aggressively optimizing the production. The trace-to-trace separation and trace width were set to 50 μm, instead of the usual more conservative value of 100 μm, in order to maximize the number of turns per layer. The number of turns was also increased by adding identical layers, 32 for the proposed design, to maximize the coil area. Since the coil surface directly affects the voltage levels generated by the coil, the coil was designed to achieve the accuracy requirements for the measurement of field harmonics, ensuring peak signals generated by the coil at the 500 mV level for a quadrupole flux density of the order of 4 T, at the maximum measurement radius, and a rotational frequency of approximately 1 rev/s.

### 3.2. Shaft and Support Structure Design

The PCB coil array is inserted in a cylindrical shaft that allows rotation around its longitudinal axis. The shaft was realized with a carbon fiber tube, produced by Refitech, readily available on the market at a very low cost, in a wide range of lengths and thicknesses. Besides being non-magnetic, the carbon fiber composite has an attractive $\frac{E}{\rho }$ ratio, around $25\times 10^{12}\;m^{2}s^{-2}$ (i.e., around twice as high as the more traditionally used fiber glass). This material is not a perfect insulator, having a resistivity of around 10 μΩm. However, an extensive series of tests done at CERN with diameters up to 100 mm and rotational speeds up to 10 turns/s showed no impact of eddy currents on the accuracy of the measured harmonics [19]. With respect to more typical designs based on custom tubes or tubular shells, fabricated on-demand to very tight tolerances but correspondingly expensive, the innovative aspect of our design consists in inserting the PCB coil array directly inside a commercial tube. The observed dimensional consistency of the units chosen is ø26±0.05 mm over a batch of 5 units.

**Figure 3:**
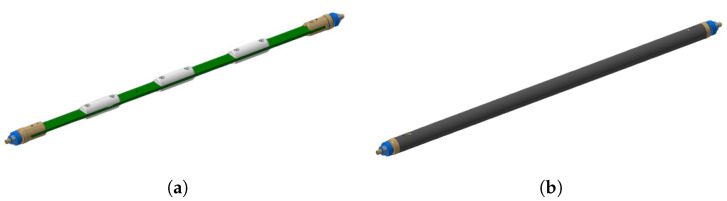
(**a**) PCB array connection head and spacer design for shaft mounting. (**b**) Carbon fiber external shaft housing the PCB array.

**Figure 4:**
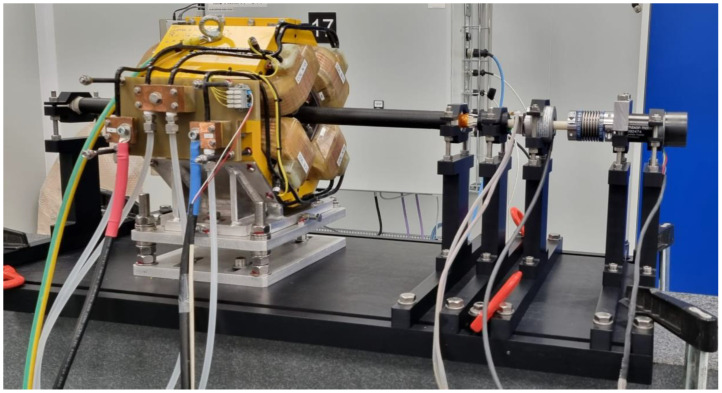
The ø26 mm rotating-coil system installed in the BTF7 quadrupole magnet used as measurement reference between CERN and INFN laboratories.

The PCB is installed at the center of the tube using semi-cylindrical centering spacers. Two connection heads are installed at the extremities of the tube. All connection and positioning pieces are 3D-printed in Accura 25^®^ plastic, leading to fast and cost-effective design modifications and reproducibility. Connection head and spacer dimensions were easily modified to perfectly adapt to the PCB’s final thickness. Adjustable support platforms and vertical V-shaped stages were designed to suspend the whole rotating system in the magnet aperture, such that the shaft rotation axis is in the mechanical center of the quadrupole magnet. A 3D view of the PCB coil array, equipped with connection heads and spacers, and of the assembled carbon fiber shaft is shown in Figure 3. The shaft rotation is realized using a commercial sensor-less DC brush-less motor, a Maxon EC 45 Flat [27]. The motor allows rotation of the shaft in both directions and, therefore, a reduction in systematic errors in measurements, by averaging results obtained in both rotation directions. A programmable high-resolution incremental encoder, Baumer EIL580P-T (maximum 65,536 optical pulses per revolution, providing a resolution of 48 μrad) [28], provides information on the angular shaft position during rotation, generating two square-wave signals, whose angular shift identifies the rotation direction, and an index square-wave signal used to identify a complete rotation of the shaft. A through-hole slip-ring is used to transmit voltage signals to the acquisition system during the continuous rotation of the shaft. The assembled rotating shaft is shown in Figure 4.

### 3.3. Control and Acquisition System Design

A data acquisition system, USB-6366 [29] from National Instruments, with a 16-bit ADC, eight independent differential analog inputs, 24 digital I/O ports, and a maximum sample rate of 2 MS/s per channel, was used to acquire all analog and digital signals. The software application for the initialization of the measurement task (specifying the acquisition channels, the acquisition parameters, and the rotation settings), the acquisition of encoder output digital signals and coil voltages, and the automatic output file generation was developed in the LabVIEW^®^ framework. Post-processing, including flux integration and harmonic analysis, was carried out with Matlab^®^ instead.

**Figure 5:**
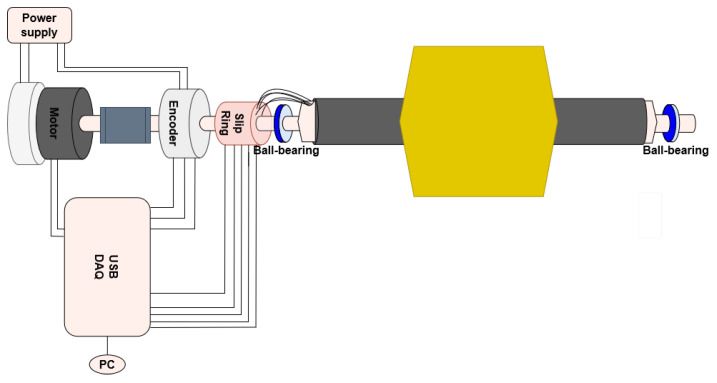
Control diagram for the proposed ø26 mm rotating-coil measurement system. A software application allows the user to configure the rotation parameters, start the acquisition of encoder and slip-ring signals, and perform the harmonic analysis.

While the coil rotates, the induced voltages in the five coils are recorded synchronously on separate channels of the ADC. In a quadrupole magnet, where the field harmonics of interest for beam control typically do not go above the order n=6 (dodecapole), the useful signal bandwidth at the maximum speed of 2 rev/s will be limited to 24 Hz. As higher-order field harmonics tend to vanish at 1/n, they can be considered as noise components, and the sampling frequency is set to 200 kS/s to leave a wide margin to prevent aliasing. At the same time, the encoder outputs are acquired as TTL signals on the digital input pins. For this application, the number of encoder pulses per revolution was set to 512, and the digital sampling frequency was set at 2 MS/s to maximize the accuracy of the pulse edge detection. It is important to remark that, since only the lowest harmonics have physical relevance, forcing the number of pulses per revolution to be a power of two has little practical impact, as long as the correct DFT or FFT algorithm is used to derive the spectral components in the post-processing phase. The voltage between two encoder pulses is integrated, yielding a measure of the linked flux between the two shaft angular positions. Mathematically, this corresponds to a re-parameterization of the signal with respect to the rotation angle instead of time. The integration is performed via software to replace expensive and dedicated digital integrator cards. The control diagram of the measurement system is shown in Figure 5.

### 3.4. Measurement Procedure

The measurement begins with the manual alignment of the shaft within the magnet aperture in correspondence with the mechanical center. Afterward, the magnet is powered, the excitation current is set at the desired level, and the shaft motor is started. Storage of the acquired signals is enabled upon reception of 5 trigger pulses on the reference channel of the encoder, in order to stabilize the shaft rotation speed before saving the acquired signals. A variable number of complete rotations, usually ten or more, is then saved in a single output file, to allow the averaging of the results to evaluate and suppress random noise components. Five voltages from the five coils, respectively, are acquired, together with the encoder pulses. Two voltages are considered: an *absolute* voltage, used to evaluate the main field component, and a *compensated* voltage, used to evaluate the higher-order harmonics. The absolute voltage is the output of one of the two outermost coils in the array. The compensated voltage, which provides the highest signal level in any field multipole of order n≤2, is a linear combination of the five coil outputs, with the intent of compensating for the main field harmonic and reducing the effect of assembly errors, mechanical vibrations, and displacements. The main harmonic compensation, also called *bucking*, can be realized by inter-connection of the coil terminals (analog bucking) and acquiring their combination, or numerically, by the individual acquisition of the coil signals (digital bucking). For the proposed system, digital compensation has been adopted (see Section 4.5) to increase the system’s flexibility, correcting possible small differences between the coils and extending the range of possible compensation schemes. At each completed revolution, the data are post-processed according to the following steps.

#### 3.4.1. Voltage Integration

**Figure 6:**
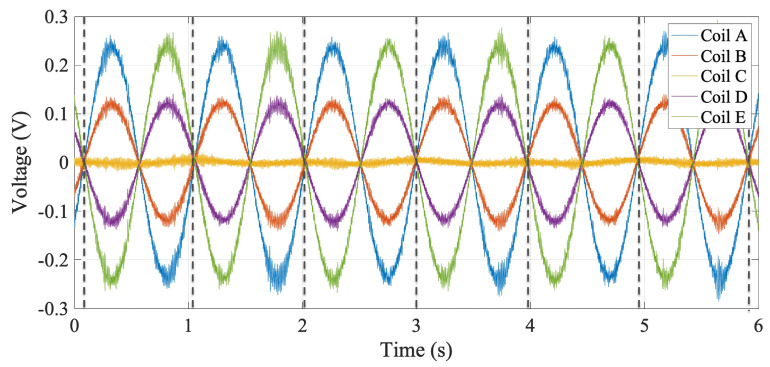
Example of acquired coil row voltages over 6 consecutive rotations. The signal amplitude is proportional to the offset of the coil rotation axis to the magnetic axis of the quadrupole magnet.

Figure 6 shows an example of raw voltages. The noise, which is approximately 2μV RMS, is partly electrical and partly mechanical in origin. The low-frequency noise component, giving rise to integrator drift, is treated in the next section. The high-frequency components are naturally reduced by integrating the signal to obtain magnetic flux, which is then used as the basis to derive magnetic field harmonics. Integration is achieved by re-parameterization of the signal as a function of the angular position, which additionally eliminates the impact of torsional vibrations. The coil voltages are integrated between consecutive encoder triggers using the trapezoidal rule. Since the encoder triggers are asynchronous with respect to the ADC time-base, the first and last analog samples of each interval are linearly interpolated to ensure respect of the integration bounds.

#### 3.4.2. Drift Correction

Integration drift is a side effect occurring when a signal is integrated, arising from the magnification of low-frequency noise components [30]. The integrated voltage signal, namely the magnetic flux, presents a linear trend in a first-order approximation. Considering that the flux change over an entire shaft rotation must be equal to zero, the drift is calculated as the remaining integrated flux after a complete shaft rotation and subsequently compensated. If the magnetic field to be measured is constant, the output voltage of the five coils is periodic. Since the acquisition electronics are in a reasonably stable environment, and given the low period of the signal (max. 2 s), the voltage offset can be assumed constant over a complete revolution. The estimated remaining flux after a complete rotation was of the order of a few mVs for the absolute signal before correction.

#### 3.4.3. Assessment of the Complex Harmonic Coefficients

The DFTs of the fluxes are computed using the Fast Fourier Transform (FFT) algorithm. The complex field harmonic coefficients are then computed using Equation (11), where the ${\underline{S}}_{n}$ values are calculated using the coil sensitivity coefficients, obtained by Equation (10), plugging in the calibrated values of area and radius.

#### 3.4.4. Feed-Down Correction

**Figure 7:**
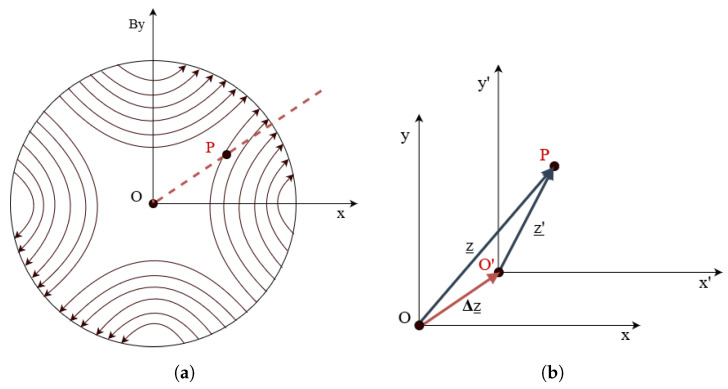
(**a**) Magnetic field lines in a normal quadrupole magnet ($A_{2}=0$). (**b**) The coordinate system with origin in O${}^{\prime }$ is used for harmonics calculation if the measurement coil rotation axis is displaced by Δz from the magnetic center, marked with O.

The feed-down phenomenon is the appearance of spurious field harmonic errors of order n<m, generated by each harmonic of order *m* when the reference system is translated. In an ideal quadrupole $C_{2}$, the magnetic field magnet is zero at the magnetic center (point O in Figure 7a). Ideally, the magnetic center is coincident with the mechanical center. If the rotating coil is placed off-axis, i.e., the measurement coil frame origin O${}^{\prime }$ in Figure 7b is displaced by Δz from the magnetic center, a dipole component $C_{1}$ proportional to the displacement will appear. The offset can be calculated from the measured harmonics as

(13) $$\Delta z=-r_{0}\frac{C_{1}}{C_{2}},$$

where Δz=Δx+iΔy.

The measured harmonic coefficients can then be corrected using the expression

(14) $$C_{n}^{{}^{\prime }}=\sum_{k=n}^{\infty }\left(\frac{(k-1)!}{(n-1)!(k-n)!}\right)C_{k}.{\left(\frac{\Delta z}{r_{0}}\right)}^{k-n}.$$

#### 3.4.5. Evaluation of Main Field Module and Phase

The main field module is obtained from the normal and skew components of $C_{2}^{{}^{\prime }}$, as taken from the absolute signal,

(15) $$|C_{2}^{{}^{\prime }}|=\sqrt{B_{2}^{{}^{\prime }2}+A_{2}^{{}^{\prime }2}}$$

and the main field direction $\alpha_{2}$ is obtained from the field phase $\phi_{2}$ as

(16) $$\alpha_{2}=\frac{\phi_{2}}{2}.$$

#### 3.4.6. Rotation and Normalization

The phase of the harmonic coefficients is based on the position of the encoder index, which is, in principle, arbitrary due to mechanical tolerances. The absolute and compensated harmonic coefficients are rotated such that the measured main field corresponds to the known type of the magnet under test; in our case, this is defined as a normal quadrupole, hence $A_{2}$ = 0. The measured harmonic coefficients are rotated by the field direction angle $-\alpha_{2}$ as

(17) $$C_{n}^{{}^{\prime \prime }}=B_{n}^{{}^{\prime \prime }}+iA_{n}{}^{\prime \prime }=C_{n}^{{}^{\prime }}e^{-in\alpha_{2}}.$$

The last step of the post-processing is the normalization of the harmonic coefficients with respect to the main field module:

(18) $$\begin{array}{cccccc} a_{n} & =10^{4}\frac{A_{n}{}^{\prime \prime }}{|C_{2}^{{}^{\prime \prime }}|}, & b_{n} & =10^{4}\frac{B_{n}{}^{\prime \prime }}{|C_{2}^{{}^{\prime \prime }}|}, & c_{n} & =10^{4}\frac{C_{n}{}^{\prime \prime }}{|C_{2}^{{}^{\prime \prime }}|}. \end{array}$$

## 4. Experimental Results

The accuracy of rotating-coil magnetometers depends on many factors. One significant contribution is given by sensitivity factors, mainly dependent on the knowledge of the coil area and rotation radius. These quantities are obtained by calibration against a reference field (area) and a field gradient (radius). Area calibration is typically performed prior to the assembly of the magnetometer to verify the quality of the coil production and identify defects such as inter-turn short circuits, which are difficult to detect through resistance measurements. Calibrating the rotating radius of each coil requires the final ball bearings to provide the final rotation axis for the best accuracy; therefore, it should performed on the fully assembled probe. In addition to the calibration, a basic mechanical characterization was carried out to evaluate the leading indicators of rotation quality and acquisition, such as loose contacts, misaligned mechanics, and vibrations.

### 4.1. Magnetic Surface Calibration

The surface calibration was performed at a field of 1 T in a reference dipole magnet available at CERN, having a relative spatial uniformity of the magnetic flux density of 200ppm. The reference field was measured using a Nuclear Magnetic Resonance (NMR) magnetometer. The calibration procedure consists of flipping the coil inside the reference magnet while integrating the voltage induced by the change in the intercepted flux. The average magnetic field B, measured by NMR along the coil length, was used as a reference field value. The voltage integrated while flipping the coil between time instants $t_{0}$ and $t_{f}$ is calculated from Faraday’s induction law as

(19) $$\int_{t_{0}}^{t_{f}}U\left(t\right)\;dt=\int_{t_{0}}^{t_{f}}-\frac{d\Phi (t)}{dt}=\Phi \left(t_{0}\right)-\Phi \left(t_{f}\right)=\Delta \Phi \left(t\right).$$

As $\phi_{1}=0$ and $\phi_{2}=\pi$, the magnetic calibrated coil surface *A_m_* can be obtained from

(20) $$\Delta \Phi \left(t\right)=A_{m}Bcos\theta_{m}-A_{m}Bcos\theta_{m-1}=2A_{m}B$$

**Figure 8:**
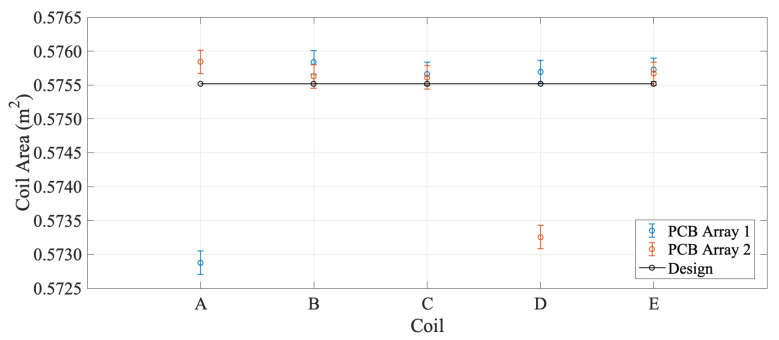
Results of coil surface calibration for PCB Array 1 and PCB Array 2, compared to the area design value.

The magnetic surface calibration was performed on two PCB arrays. The results are compared to design coil area $A_{d}$ in Figure 8 and reported in Table 5, along with the relative difference $\Delta =\frac{A_{d}-A_{m}}{A_{d}}$.

### 4.2. Coil Radius Calibration

The calibration of the coil radii was carried out in the BTF7 quadrupole magnet, used as a reference. The calibration of the coil radii requires knowledge of the field gradient. A measurement performed by a Single-Stretched Wire (SSW) system was used as a reference. The integrated gradient *g* over the magnetic length $L_{m}$ is defined as

(21) $$g=G_{c}L_{m}=\int_{-\infty }^{+\infty }G\left(l\right)\;dl$$

where $G_{c}$ is the field gradient measured along the wire displacement, and G(l) is the field gradient as a function of the position.

From Equation (10), the sensitivity coefficient ${\underline{S}}_{2}$ to the quadrupole component (obtained for ${\underline{z}}_{1}=x_{1}$ and ${\underline{\zeta }}_{2}=x_{2}$ in Figure 1) is given by

(22) $$\begin{array}{cc} S_{2} & =\frac{NL}{2r_{0}}\left[{\left(x_{2}+iy\right)}^{2}-{\left(x_{1}+iy\right)}^{2}\right]=\frac{NL}{2r_{0}}[\left(x_{2}^{2}-x_{1}^{2}+i2y\left(x_{2}-x_{1}\right)\right]\\ \; & =\frac{NL}{r_{0}}w\left(r_{c}+iy\right)=\frac{A_{c}}{r_{0}}\left(r_{c}+iy\right) \end{array}$$

where $w=x_{2}-x_{1}$ is the coil width, $r_{c}=\frac{x_{1}+x_{2}}{2}$ is the average radius of the absolute coil winding, and *y* is the coil’s vertical displacement from the PCB array plane. From Equation (21) and Equation (22), and considering the definition of the gradient as $G=B_{2}/r_{0}$, the average coil radius is obtained as

(23) rc=Re(S2Acr0)=r0AcRe(Ψ2C2)=r0Ac(Re(Ψ2)B2)=r0AcRe(Ψ2)Gr0=1AcRe(Ψ2)G=LmAcRe(Ψ2)g

The nominal values for coil rotation radius $r_{d}$ and the calibrated rotation radius $r_{c}$ are reported in in Table 6, along with the calibrated radius $r^{*}$ relative to the central coil C taken as a reference, for each coil on PCB Array 1. The difference $\Delta =r_{d}-r^{*}$ from nominal design values is in the order of tens of μm.

### 4.3. Mechanical Characterization

**Figure 9:**
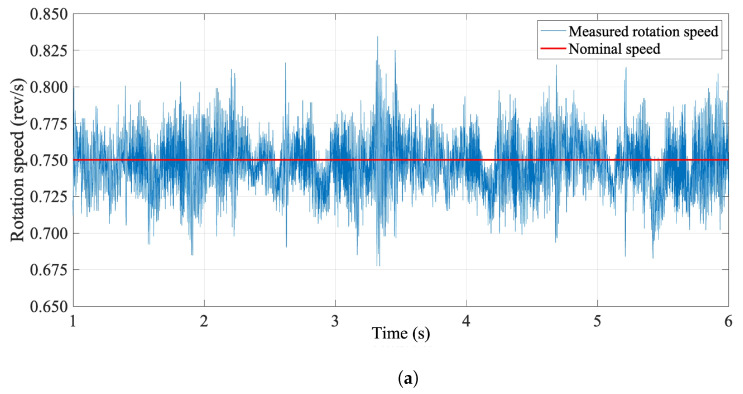

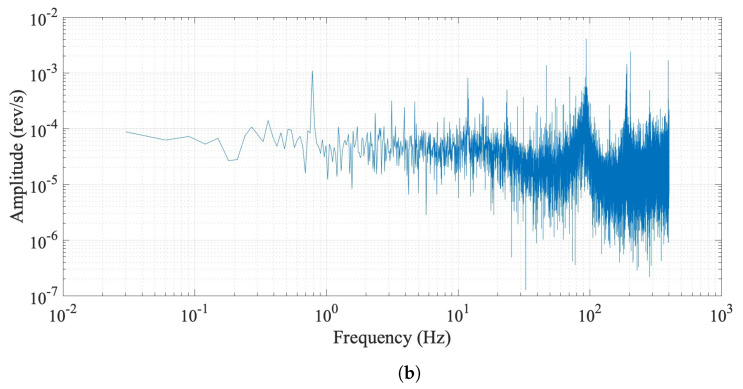
(**a**) The instantaneous rotation speed signal in the time domain. (**b**) Frequency spectrum of instantaneous rotation speed signal at 0.75 rev/s nominal speed.

In order to characterize the robustness of the system against torsional vibrations, the rotational speed was obtained from timing intervals between encoder pulses for different rotation speed levels over 100 consecutive rotations. The results are reported in Table 7 in terms of the measured mean rotation speed $\bar{\omega }$ and standard deviation σ, as well as the RMS and peak-to-peak of their relative difference in percentage. The presence of modal vibration components was also investigated by analyzing harmonic components in the speed spectrum, reported for a nominal speed value of 0.75 rev/s in Figure 9. The spectrum resembles, as expected, a flat white noise distribution, with most components below the $10^{-3}$ rev/s level. The low-frequency peak at 0.75 Hz is the fundamental component associated with the rotation speed, while the resonance at around 90 Hz appears to be linked to the lowest torsional mode of the shaft.

**Figure 10:**
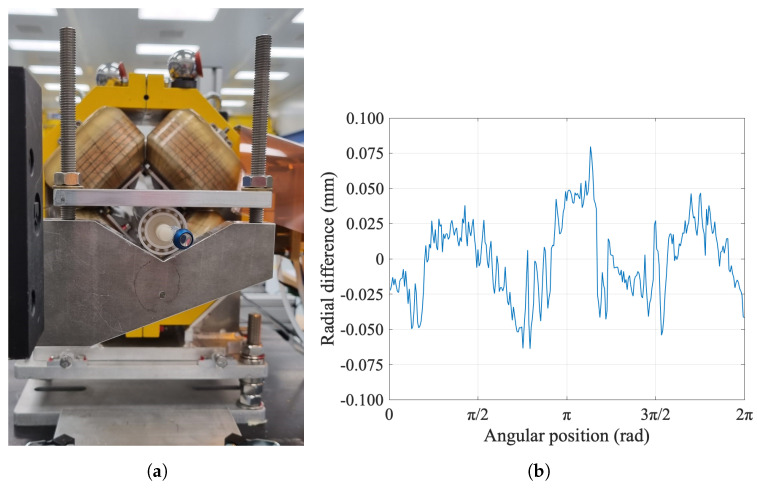

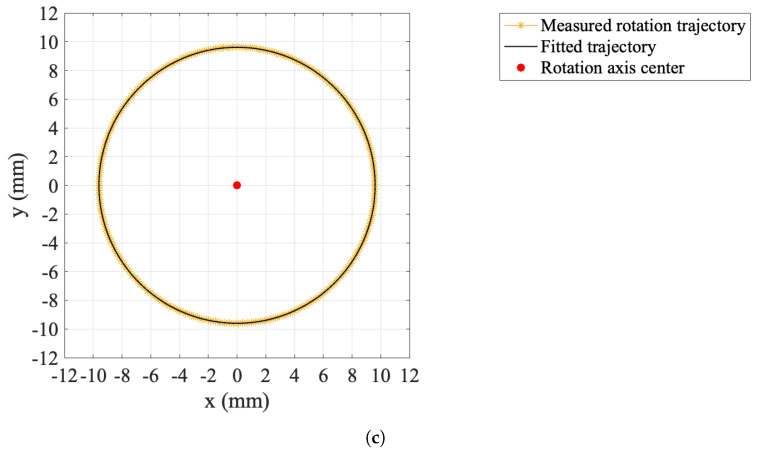
(**a**) Retroreflector positioned at one shaft end for rotation axis localization. (**b**) Laser tracker measurements fit by the ideal circular trajectory. (**c**) Radial fit difference of the fitted circle to the laser tracker measurements. The results were obtained over 25 consecutive turns.

The mechanical quality of the rotation was further investigated by using a laser tracker to acquire a set of points from a retro-reflector mounted at one shaft end, as shown in Figure 10a, while rotating at the nominal speed of 0.75 rev/s. The measured points were then fitted to a circle, as shown in Figure 10b. The center of this ideal circle, arbitrarily set to the origin of the coordinate system, represents the rotation axis of the coil shaft. The absolute value of the radial difference between the measured points and the ideal circle is below 80 μm, as shown in Figure 10c as a function of the rotation angle. The impact of this imperfect rotation is mitigated by bucking the coil signals used for the measurement of field harmonics. The rotation axis is offset by Δx = −0.522 mm and Δy = −0.856 mm with respect to the geometrical axis of the carbon fiber tube, obtained by moving by hand the retro-reflector all over the outer surface of the tube. Since the axis of rotation of the PCB coil array is defined by the connection heads at the extremities, rather than the tube, this offset does not affect the measurement quality.

### 4.4. Magnetic Loadline

**Figure 11:**
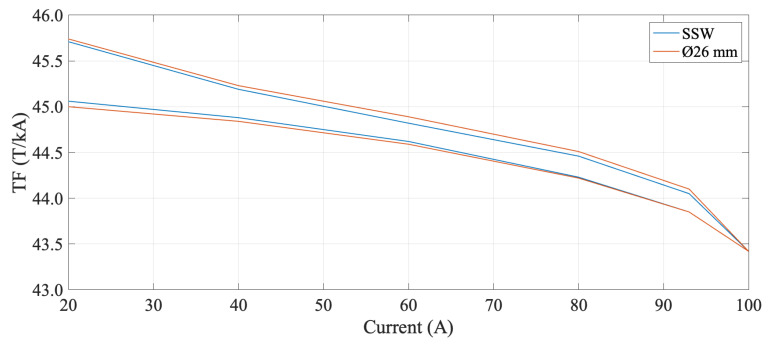
Magnet transfer function measured in DC mode from 0 to 100 a and back to 0, compared to the result obtained with Single Stretched Wire system. For each current plateau, the averaged gradient at the magnet center was divided by the corresponding current value. The slope of the curve is due to saturation of the iron yoke.

The magnet Transfer Function (TF), which is the ratio between the field and excitation current, was measured in DC mode on current plateaus from 0 A up to 100 A and compared with the results obtained by a Single Stretched Wire (SSW) measurement system. The magnet Transfer Function is shown in Figure 11 and is in good general agreement with the Single Stretched Wire measurements. The integrated gradient at the nominal magnet current of 93 A, computed after magnet pre-cycling, is −4.0789 T, i.e., equal (by calibration) to the reference value measured with the SSW, with a repeatability $\sigma =6.6391\;10^{-5}$ T.

### 4.5. Bucking Ratios

**Figure 12:**
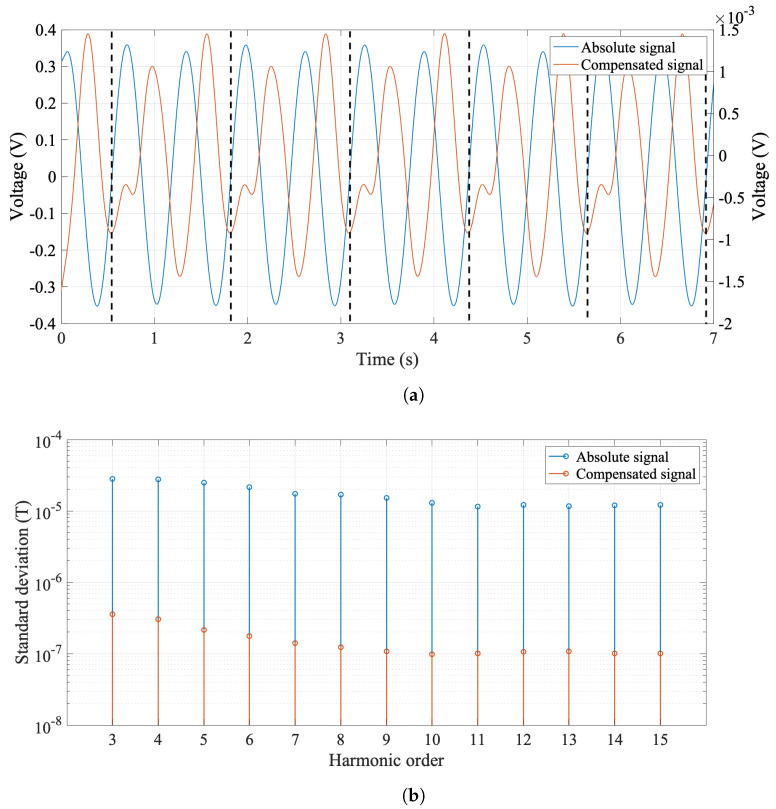
(**a**) Absolute and compensated filtered voltage signals for 5 consecutive rotations (single rotation is between two dotted lines) at 0.75 rev/s nominal speed. (**b**) Standard deviation for higher-order harmonic coefficients $C_{n}$, computed from the absolute and compensated signal. The results were obtained at $r_{0}$ = 10 mm, nominal current 93 A, and computed over 25 rotations.

The number of coils in the PCB array depends on the compensation scheme necessary for the magnet to be tested, and it is chosen during the design phase. Mechanical imperfections (due to assembly errors) and rotation errors (vibration and torsion) degrade the metrological performance, giving rise to spurious harmonic content [9]. Since all coils on the PCB array are nominally identical and subjected to the same vibration modes, compensation is achieved by taking a suitable linear combination of the outputs of two or more individual coils. Compensation of the first two components of the field is required to achieve sufficient accuracy in measuring the harmonic content of a quadrupolar field. As reported in Section 3.4, this process is commonly referred to as *bucking*. In the adopted digital bucking configuration, $Coil_{E}$ is used to measure the most dominant harmonic term. The dipole and quadrupole bucked signal is then digitally constructed according to the following linear combination: $Coil_{E}-Coil_{D}-Coil_{C}+Coil_{B}$. The acquired voltage signals in the absolute and compensated configurations are shown in Figure 12a. The advantages of the compensation scheme are visible in Figure 12b, showing lower accuracy in the measurement of the harmonic coefficient from the absolute signal. The ratio of the second harmonic coefficient (quadrupole) in the absolute and compensated measurements is called the compensation or bucking factor, and is around 700.

### 4.6. Comparison with an Existing Rotating-Coil System

**Figure 13:**
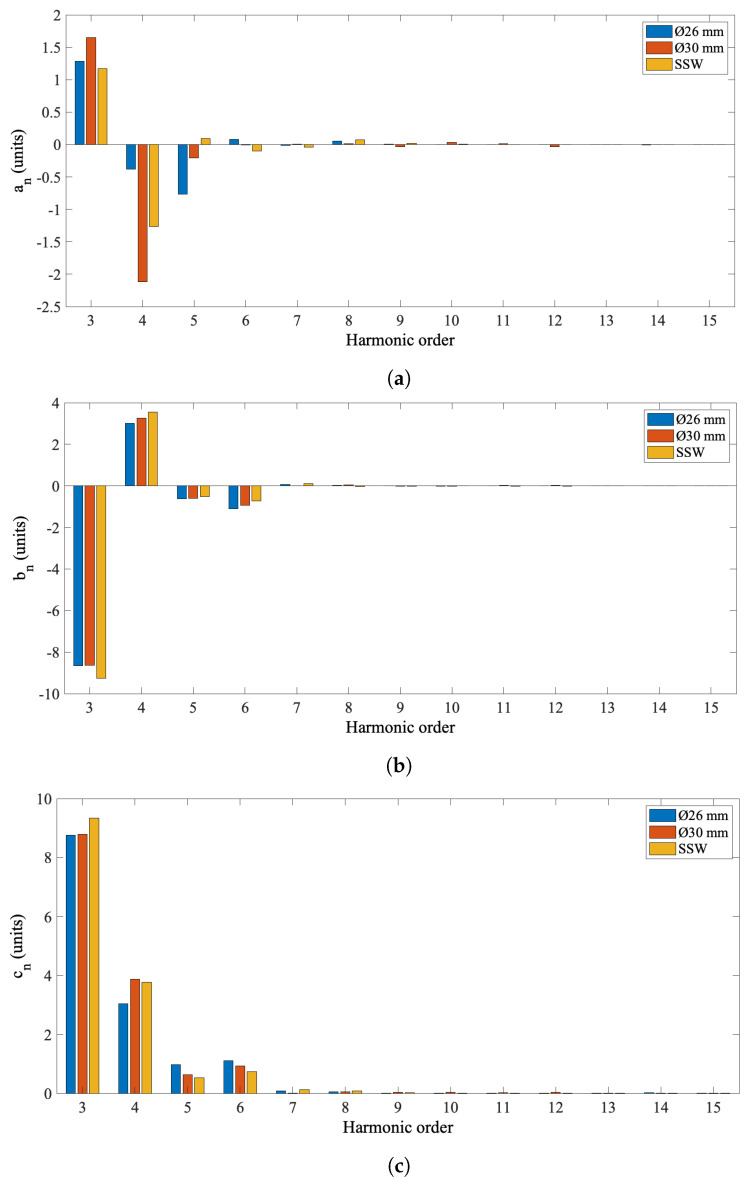
(**a**) Higher-order normalized skew coefficients $a_{n}$. (**b**) Higher-order normalized normal coefficients $b_{n}$. (**c**) Higher-order normalized harmonic coefficients $c_{n}$. Higher-order coefficients are expressed at $r_{0}$ = 10 mm and nominal current 93 A and measured with both ø26 and ø30 mm rotating-coil systems and SSW system. Rotating-coil results have been averaged over 100 revolutions, at the nominal rotation speed of 0.75 rev/s.

**Figure 14:**
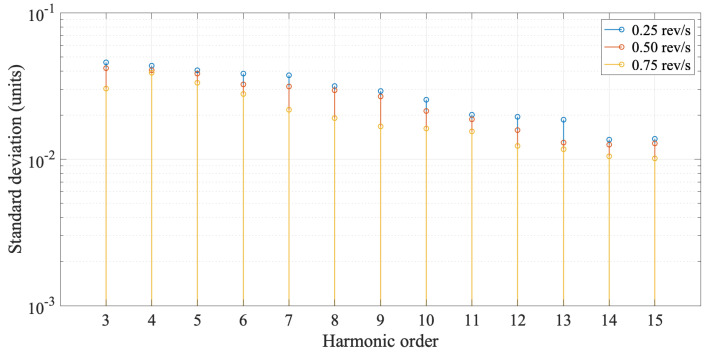
Comparison of measurement repeatability for higher-order multipoles $c_{n}$ at $r_{0}$ = 10 mm and nominal current 93 A for different rotation speed values.

A ø30 mm rotating-coil probe, previously in use at CERN, was used to test the same quadrupole magnet for the cross-checking of the ø26 mm rotating-coil system, in terms of measured harmonic field components. Measurements were performed adopting two pre-cycling conditions: (i) after degaussing, (ii) pre-cycling the magnet with three cycles between 0 and 100 A. The ø30 mm rotating-coil measurement system includes a fast digital integrator [31] for voltage integration and a software application to control the hardware components and for the acquisition and post-processing of data. The field multipole measurement results obtained from the quadrupole compensation scheme are reported in Figure 13 and compared to the results obtained from the ø30 mm system and the SSW system. Higher-order harmonics computed over 100 repetitions and measurement repeatability are reported in Table 8, compared to measurement results obtained with the ø30 mm rotating-coil system. The measurement repeatability was tested at different rotation speed values and the results reported in Figure 14 show the same pattern for the standard deviations.

## 5. Discussion

The PCB technology adopted for coil production allowed high control over track positioning, albeit with some issues. Both Coil A in PCB Array 1 and Coil D in PCB Array 2 are missing around 0.4% of the coil area, as reported in Table 5. This can be attributed in all likelihood to a single inter-turn short circuit, which was found, however, not to be straightforward to identify even with a four-wire measurement. PCB Array 1 was selected for the probe, excluding Coil A from the analysis. Considering only the four remaining good coils on each array, the nominal and calibrated coil areas differ from the design value by around 160 ppm RMS. The measurement repeatability is as low as 0.6 ppm. The calibrated coil radii $r^{*}$ in Table 6, computed with respect to the symmetry axis of the central coil *C*, differ from the design value by around 35μm RMS, i.e., 0.35% relative to the outer coil radius. The repeatability of the radius measurement is 0.03%. The reason for this poor result is that the computation of the radius, according to Equation (22), combines the errors in both the coil area and the magnetic flux measurements. Moreover, there appears to be a difference between the calibrated radii on one side of the PCB, i.e., for Coils D and E, which are very close to their nominal values, and those of Coils A and B, which have an error of up to 67μm. This asymmetry could be due to a systematic effect in the PCB manufacturing, which will be investigated further on the other PCBs of the same batch. In conclusion, for a target accuracy of the rotating-coil system measurement in the range of 100ppm, the nominal values of the geometrical parameters are not sufficient and the magnetic calibration procedure is indispensable.

The mechanical characterization allowed us to choose the optimal rotation speed as a trade-off between the coil output voltage amplitude and vibration effects. Results show speed uniformity less than 1.5%, independent of the speed level, and a peak-to-peak variation of less than 10% at 0.25 rev/s and decreasing at around 5% at 0.75 rev/s. The rotation speed was set at 0.75 rev/s, obtaining at the nominal current of 93 A output voltages in the range of 1 V_pp_. Higher rotation speed values would increase the amplitude of the induced voltages, but were observed to cause noticeable flexural vibrations and were therefore discarded. The higher-frequency torsional components observed in the speed spectrum in Figure 9 have no effect on the measurement results.

The field homogeneity of the BTF7 quadrupole was measured at the nominal current value of 93 A and the results are expressed in terms of magnetic multipoles in units of $10^{-4}$ at a reference radius of 10 mm with respect to the main field in Table 8 and Figure 13 for field errors. The relatively high values of harmonics $c_{3}$ and $c_{4}$ are related to the difficulties in respecting the mechanical tolerances during magnet assembly. The results highlight the effectiveness of digital bucking of the dipole and quadruple field components in reducing the standard deviation of higher-order multipoles by almost two orders of magnitude, down to well below 3μT, i.e., around 1 ppm.

## 6. Conclusions

In this article, the design and development of a new small-diameter rotating-coil measurement system prototype, needed to test the field quality of small-diameter (30 mm or higher) bore quadrupoles for the European project EuPRAXIA, is presented. Accurate measurements of the field harmonics in accelerator magnets require the construction of a dedicated rotating-coil shaft of the largest possible diameter. The proposed magnetometer design is based on an array of identical coils realized in PCB technology and mounted in a cylindrical shaft with a diameter of ø26 mm.

Some considerations about the manufacturing process were reported. Excluding from analysis two defective coils, the accuracy of track positioning on the PCB can be evaluated to be around 2μm. This is an impressive value for a 32-layer PCB with the aggressively optimized parameters listed in Table 4, and it is the basis of the excellent measured quadrupole bucking ratio of approximately 700. However, if one considers the small size of the coils, this tolerance still leads to peak calibrated rotation radius errors up to 0.5%, which are unacceptable for the characterization of accelerator beam line magnets. This underlines the importance of the magnetic calibration for high-accuracy applications. In the future, optical checks to identify faulty coils shall be carried out systematically on each individual layer.

The results obtained from the mechanical characterization essentially validate the innovative mechanical design, which is based on inserting the PCB coil array in a carbon fiber tube by means of 3D-printed plastic inserts. Despite the low rigidity of the Accura 25 ^®^ used for the inserts, and the need to retouch them slightly on the lathe, the quality of the rotation is very good, with torsional vibrations peaking at the safely high frequency of 90Hz and within 1.5%RMS in amplitude. Resorting to low-cost, commercially available components and 3D-printed parts is key to ensuring the wide applicability of this design, as well as fast and cost-effective modifications.

Comparison with other measurement instruments proved that our prototype rotation coil system can achieve an absolute accuracy of the main integrated gradient of approximately 50ppm, with a repeatability of 10ppm, while the accuracy of high-order compensated harmonics is approximately 100ppm with a repeatability of 10ppm. This performance meets the requirements for the measurement of the EuPRAXIA magnets.

Commissioning this prototype system has provided valuable experience to progress in the design of the production instrument. First of all, different 3D-printed materials and techniques will be tested for the inserts and the shaft end connections, with the goal of minimizing or avoiding completely the need for further mechanical retouches. The set of screws and pins that lock the PCB and the inserts in place inside the carbon fiber tube should also be redesigned, to facilitate the disassembly process, as this may be needed for maintenance. Finally, the shaft support system shall be augmented with the possibility of shifting horizontally the instrument within the magnet bore, so as to allow one to improve the accuracy of the in situ radius calibration procedure, as described in [32]. The final rotating-coil system shall be used at CERN to complete the characterization of the BTF7 quadrupole, including, in particular, the measurement of the magnetic axis offset with respect to the magnet’s fiducial reference systems. This measurement requires the addition of suitable, reproducible optical targets to the rotating coil, which were missing in the prototype but shall be added to the final system.

## Figures and Tables

**Table 1:** Specifications of the BTF7 EuPRAXIA quadrupole prototype tested at CERN.

Parameters	Values
Aperture diameter	ø45 mm
Reference radius $r_{0}$	10 mm
Nominal magnetic field gradient	50 T/m
Nominal current	93 A
Iron yoke length	440 mm
Total field extension (incl. fringe)	600 mm

**Table 2:** Measurement accuracy specifications for characterization of EuPRAXIA quadrupole magnets.

Parameters	Accuracy
Integrated gradient	100 ppm
Field harmonics @ $r_{0}$	100 ppm
Magnetic axis	150 μm

**Table 3:** PCB manufacturing parameters of a coil array prototype.

Manufacturing Parameters	Value
Copper track thickness	12 μm
Copper track width	50 μm
Copper track distance	50 μm
Via diameter	25 μm
Internal pad width	25 μm
External pad width	50 μm
FR-4 sheet thickness	50 μm
Pre-preg sheet thickness	50 μm

**Table 4:** Design parameters of the coil prototype.

PCB design parameters	Value
PCB width	25.5 mm
PCB total length	620 mm
PCB thickness	3.2 mm
**Array design parameters**	**Value**
Array width	25 mm
Array length	601.45 mm
Total number of coils	5
**Coil design parameters**	**Value**
Coil inner length	600.25 mm
Coil inner width	3.5 mm
Coil design area	0.5757 m${}^{2}$
Number of layers	32
Turns per layer	8
Total number of turns	256

**Table 5:** Calibration results for coil area in PCB Array 1 and PCB Array 2.

PCB Array 1	Coil A	Coil B	Coil C	Coil D	Coil E
$A_{d}$ [m${}^{2}$]	0.5757	0.5757	0.5757	0.5757	0.5757
$A_{m}$ [m${}^{2}$]	0.5728	0.5758	0.5756	0.5756	0.5757
Δ [ppm]	−4990	149	−153	−97	−45
**PCB Array 2**	**Coil A**	**Coil B**	**Coil C**	**Coil D**	**Coil E**
$A_{d}$ [m${}^{2}$]	0.5757	0.5757	0.5757	0.5757	0.5757
$A_{m}$ [m${}^{2}$]	0.5758	0.5756	0.5756	0.5732	0.5756
Δ [ppm]	162	−213	−239	−4431	−147

**Table 6:** Calibration results for PCB Array 1 coil rotation radius.

PCB Array 1	Coil A	Coil B	Coil C	Coil D	Coil E
$r_{d}$ [mm]	9.500	4.750	0	4.750	9.500
$r_{c}$ [mm]	9.460	4.799	0.028	4.721	9.472
$r^{*}$ [mm]	9.432	4.772	0	4.750	9.500
Δ [μm]	67.6	21.8	0	0.5	0.0

**Table 7:** Rotation speed analysis results.

Parameter	0.25 [rev/s]	0.50 [rev/s]	0.75 [rev/s]
$\bar{\omega }$ [rev/s]	0.251	0.514	0.752
σ [rev/s]	4.973 × $10^{-3}$	6.316 × $10^{-3}$	5.822 × $10^{-3}$
RMS ($\frac{\Delta \omega }{\bar{\omega }}$) [%]	1.643	1.437	1.313
${\left(\frac{\Delta \omega }{\bar{\omega }}\right)}_{pp}$ [%]	8.656	6.818	5.825

**Table 8:** Comparison of measurement repeatability for higher-order multipoles $c_{n}$ at $r_{0}$ = 10 mm and nominal current 93 A measured with both rotating over 100 rotations, at the nominal rotation speed of 0.75 rev/s.

Harmonic Order	ø26 mm *c_n_*	ø26 mm σ	ø30 mm *c_n_*	ø30 mm σ
	[units]	[units]	[units]	[units]
$c_{3}$	8.75	3.03 × $10^{-2}$	8.79	2.03 × $10^{-2}$
$c_{4}$	3.04	3.88 × $10^{-2}$	3.88	2.03 × $10^{-2}$
$c_{5}$	0.98	3.32 × $10^{-2}$	0.63	4.31 × $10^{-2}$
$c_{6}$	1.10	2.78 × $10^{-2}$	0.93	4.64 × $10^{-2}$
$c_{7}$	0.06	2.18 × $10^{-2}$	0.01	5.2 × $10^{-2}$
$c_{8}$	0.03	1.91 × $10^{-2}$	0.05	1.06 × $10^{-2}$
$c_{9}$	0.02	1.67 × $10^{-2}$	0.04	6.70 × $10^{-2}$
$c_{10}$	0.01	1.55 × $10^{-2}$	0.02	9.59 × $10^{-2}$
$c_{12}$	0.01	1.53 × $10^{-2}$	0.04	1.57 × $10^{-2}$
$c_{13}$	0.01	1.47 × $10^{-2}$	0.02	3.25 × $10^{-2}$
$c_{14}$	0.01	1.45 × $10^{-2}$	0.01	2.53 × $10^{-2}$
$c_{15}$	0.01	1.43 × $10^{-2}$	0.01	2.42 × $10^{-2}$

## Data Availability

The data that support the findings of this study are available from the authors, upon reasonable request.

## Abbreviations

The following abbreviations are used in this manuscript:

SSW	Single Stretched Wire
